# Premature Burial

**Published:** 1896-04

**Authors:** 


### Editorial.

### PREMATURE BURIAL.

*The Embalmer's Monthly* remarks that more than the usual number of cases of suspended animation have been reported of late, and goes on to record in detail seven recent instances of apparent death and nearly or quite completed burial from which (except the latter) the subjects were barely rescued, as by accident.

From which it is immediately to be inferred that in order to be "good and dead" the services of an embalmer had better be secured. Such services while not absolutely essential to the complete shuffling off of the mortal coil, will certainly and surely remove all possibility of the apparent corpse being placed in an embarrassing situation at the very outset of his journey from this vale of strife.

The subject of premature burial always arouses the sharpest interest. We must all go the way of earth. "All that live must die, passing through nature to eternity."

Death is an awful certainty, of which all are more or less conscious and expectant, but the very soul shudders at the black horror of premature inhumation. The thought of the terrible, impotent struggle for breath in the confinement of the coffin under six feet of earth, with mind fully awake to the utter hopelessness of aid, and the gradual cessation of effort as the rapidly beclouding mind realizes its futility, and finally the slow yielding up of the spirit, is calculated to strike terror to the hearts of the bravest. It exceeds in weirdness the morbid tales of Poe, of the Pit and Pendulum, and the cask of Amontillado. But, like these fantastic creations, premature burial is in all probability a fiction. A careful consideration of the practical facts in connection with burial will rob death of at least one of its terrors, and at the same time put a spoke in the embalmer's wheel. No one can live for any length of time in a casket that is hermetically sealed. The supply of oxygen is rapidly exhausted, and asphyxiation necessarily follows in a very short time. Even if one were interred in a state of perfect health, death would be a matter of a few moments, and it is probably even more rapid in those cases, if they exist at all, of burial before life is extinct, or persons in the condition of suspended animation. In the latter consciousness is already absent at interment and there is no possibility of its revival while asphyxiation is taking place.

The seven instances mentioned above are doubtless mere newspaper sensations, or genuine cases of recovery from apparent death before interment, which are not rare. The custom in the North of keeping the bodies of the dead for several days before burying them, and until unequivocal signs of death are apparent, does away entirely with the present very slight risk of premature burial. In the South, the dead are buried within twenty-four hours to thirty-six hours after death, but not until it is certain that life is actually extinct.

Some States have a law that prohibits post-mortems being performed until twenty-four hours after death. Many will recall in this connection, the case of Washington Irving Bishop, the so-called mind reader, upon whom an autopsy was performed in New York some years ago. The post-mortem examination was made about twelve hours after death, and it was claimed by the family of the deceased, in a suit brought against the surgeons who performed the autopsy, that the latter was the cause of death, and that Bishop was in a condition of self induced catalepsy. It was proven definitely by the defendants that the man was unquestionably dead before the examination was begun.

Our scientific knowledge of the signs of death is quite sufficient to establish it under all circumstances, and one may safely banish from his mind the possibility of premature burial and may regard it as mere sensationalism.

